# Evaluating fine changes in visual function of diabetic eyes using spatial-sweep steady-state pattern electroretinography

**DOI:** 10.1038/s41598-023-40686-5

**Published:** 2023-08-22

**Authors:** Norihiro Nagai, Yasuaki Mushiga, Yoko Ozawa

**Affiliations:** 1 Department of Ophthalmology, St. Luke’s International Hospital, Tokyo, Japan; 2 Department of Ophthalmology, Keio University School of Medicine, Tokyo, Japan; 3 Department of Clinical Regenerative Medicine, Eye Center, Fujita Medical Innovation Center Tokyo, Fujita Health University School of Medicine, 7-16-14 Ginza, Chuoku, Tokyo, 104-8313 Japan

**Keywords:** Imaging and sensing, Retinal diseases

## Abstract

The visual function of diabetic eyes was assessed to evaluate spatial-sweep steady-state pattern electroretinography (swpPERG) as a potential high-sensitivity analysis method. Data from 24 control eyes, 28 diabetic eyes without diabetic retinopathy (DR), and 30 diabetic eyes with DR (all with best-corrected visual acuity [BCVA] better than logMAR 0.05; median age, 51) in response to spatial-patterned and contrast-reversed stimuli of sizes 1 (thickest) to 6 were converted into the frequency domain using a Fourier transform and expressed as signal-to-noise ratios (SNRs). SNRs of diabetic eyes, both with and without DR, were lower than those of controls (*P* < 0.05), and those of DR eyes were lower than those of diabetic eyes without DR (*P* < 0.05). The SNRs were correlated with ganglion cell layer volume measured using optical coherence tomography (OCT) and foveal vascular length density at the superficial retinal layer measured using OCT angiography (*P* < 0.05 or < 0.01, according to stimulus size). Therefore, swpPERG SNRs could detect fine reductions in visual function in diabetic eyes and were particularly low in DR eyes. Moreover, SNRs were correlated with inner retinal morphological changes in diabetic eyes, both with and without DR. swpPERG may therefore be useful for detecting fine fluctuations in visual function in diabetic eyes.

## Introduction

Recent advancements in medical science have led to significant improvements in the treatment outcomes of diabetic retinopathy (DR)^1,2^. However, the global prevalence of DR is expected to remain high^1^, and consequently, vision loss in the working population is also likely to be a significant social issue worldwide in the future. Early diagnosis is important for good visual outcomes^3,4^ but patients may not recognize the seriousness of the disease until their vision deteriorates significantly. Therefore, the detection of fine but relevant changes in visual function could be extremely valuable for making patients aware of their illness before substantial visual loss occurs, as well as for encouraging and facilitating treatment compliance.

Moreover, recent studies have provided proof-of-concept for several novel neuroprotective approaches that have the potential to preserve visual function^5–7^. These therapies may be valuable tools not only for preventing blindness, but also for preserving the quality of vision, which has a significant impact on the overall quality of life of patients^8^. For approval based on clinical trials, the detection of subtle but significant visual changes may be necessary. The issue related to clinical trials is that the neuroprotective intervention would be initiated before retinal neural degeneration and visual function impairment become substantial and irreversible, and the effects would be detected with high sensitivity to shorten the trial periods; given that neurodegeneration can progress gradually, the difference of the conditions after treatment may be detected only after a long time with conventional measurement systems for visual function.

In fact, before visible fundus changes appear, visual function impairment is traditionally reported as impaired oscillatory potentials, which indicate inner retinal dysfunction, recorded using conventional scotopic electroretinography (ERG)^9,10^. These parameters could be used to detect fine differences in visual function, but they need a relatively longer time for recording. Morphological changes in the retinal ganglion cell (RGC) and inner retinal layers of patients in the early stages of diabetes have been also observed using optical coherence tomography (OCT)^11–13^. However, whether there are any correlations between functional and morphological changes remains unclear.

Spatial-sweep steady-state pattern ERG (swpPERG) has been shown to be able to detect subtle visual changes and variations in healthy subjects with good BCVA^14^. swpPERG data is recorded in response to the stimulus of spatial-pattern (horizontal grating) images with contrast-reversal in time at 7.5 Hz (15 reversals per second) in a bright field. This stimulus maintains a constant space-averaged luminance that does not change over time and does not elicit a response from the retina beyond the area directly stimulated by the patterned field. Fourier transform is used to deconstruct the averaged complex waveform into its frequency components^15–17^ and responses corresponding to the 10 Hz data are expressed as the signal-to-noise ratio (SNR). Thus, the influence of noise, such as that produced by eye movements and other environmental electromagnetic sources, can be excluded from the data.

In this study, we used swpPERG to assess the visual function of healthy volunteers and diabetic patients with and without diabetic retinopathy (DR) and with good best-corrected visual acuity (BCVA), and compared it with morphological data obtained using OCT and OCT angiography (OCTA). This study will help in identifying fine changes in visual function and their relationships with morphological changes in the diabetic retina to help clarify the pathogenesis of DR.

## Results

Twenty-four control eyes, 28 diabetic eyes without DR, and 30 diabetic eyes with DR including 12 eyes with proliferative DR were analyzed (overall median patient age, 51 [range 27–61] years). There were no significant differences in age between the three groups (*P* = 0.053) (Table 1). While there were differences in BCVA among the three groups (*P* < 0.01), the BCVA of all eyes was better than 0.05 in logMAR (overall median BCVA, − 0.18 [range − 0.18 to 0.05]). As regards mean BCVA of diabetic eyes with and without DR, there was no difference (*P* = 0.389). Intraocular pressure (*P* = 0.076) and central retinal thickness (*P* = 0.932) also did not differ significantly between diabetic eyes with and without DR.

**Table 1:** Characteristics of the eyes.

	Control (n = 24)	DM with no DR (n = 28)	DR (n = 30)	*P*^a^	*P*^b^	*P*^c^
Age	44.8 (45.5) [27–61]	49.6 (50) [37–60]	52.2 (54) [42–60]	0.053	0.270	0.083
logMAR BCVA	-0.17 (-0.18) [-0.18–-0.08]	-0.12 (-0.08) [-0.18–0.05]	-0.10 (-0.08) [-0.18–0.05]	< 0.001**	0.001**	0.389
Intraocular pressure (mmHg)	13.7 (13.5) [10.3–19.0]	17.1 (17.3) [12.0–22.4]	15.8 (15.3) [11.5–21.4]	< 0.001**	< 0.001**	0.076
Central retinal thickness (μm)	221 (219) [202–243]	234 (229) [207–308]	236 (229.5) [199–311]	0.030*	0.014*	0.932

Data are presented as average, median and ranges. ^a^Kruskal–Wallis test comparing three groups, and ^b,c^Mann–Whitney U test, comparing between ^b^control and diabetic eyes with no DR, and ^c^diabetic eyes without and with DR. DM, diabetes mellitus; DR, diabetic retinopathy; BCVA, best-corrected visual acuity. *P < 0.05, **P < 0.01.

**Figure 1:**
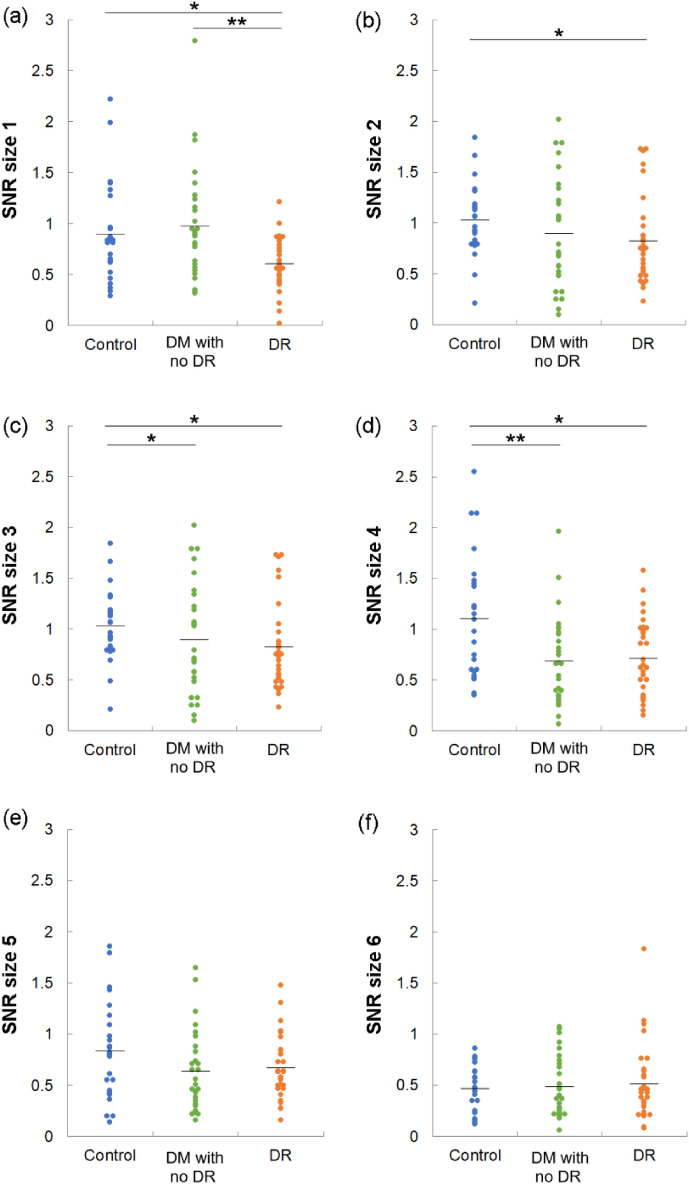
Signal to noise ratio (SNR) of control and diabetic eyes without and with diabetic retinopathy. DM, diabetes mellitus; DR, diabetic retinopathy. *P* values of Kruskal–Wallis test comparing 3 groups were (**a**) 0.006, (**b**) 0.088, (**c**) 0.044, (**d**) 0.013, (**e**) 0.296, (**f**) 0.996. *P < 0.05, **P < 0.01 by Mann–Whitney U test, comparing 2 groups.

swpPERG SNRs were recorded using contrast-reversed stimuli of sizes 1 (thickest) to 6 (Fig. 1a–f). We found that the SNRs of DR eyes were lower than those of control eyes in response to stimuli sizes 1–4 (respective *P* values for sizes 1–4, 0.032, 0.014, 0.030, and 0.013) (Fig. 1a–d). The SNRs of diabetic eyes without DR were also lower than those of control eyes for stimuli sizes 3 (*P* = 0.032) and 4 (*P* = 0.008) (Fig. 1c, d). Moreover, the SNRs of DR eyes were lower than those of diabetic eyes without DR for size 1 stimuli (*P* = 0.002) (Fig. 1a).

**Figure 2:**
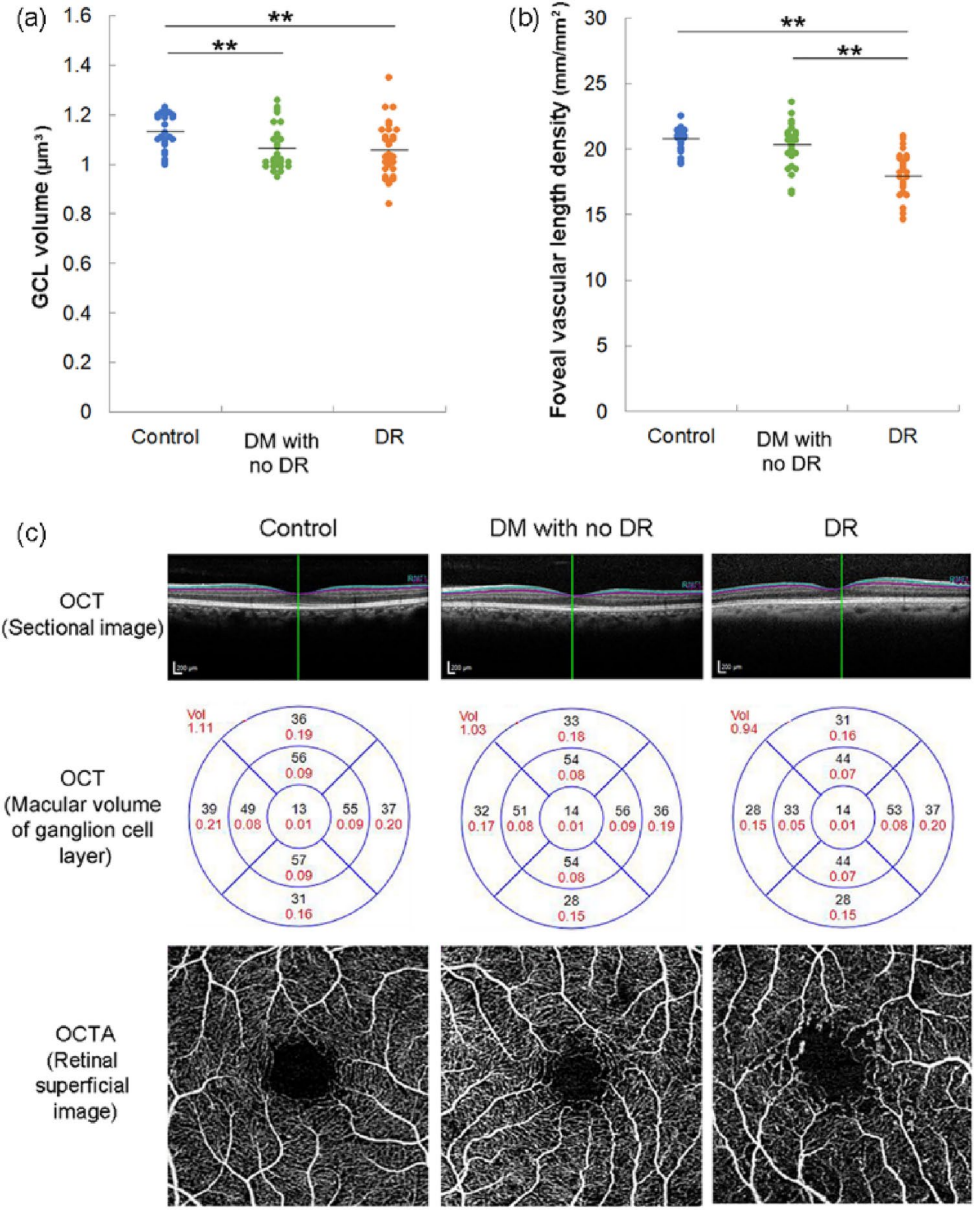
Morphological differences of control and diabetic eyes without and with diabetic retinopathy. (**a**) Ganglion cell layer (GCL) volume measured by optical coherence tomography (OCT). (**b**) Foveal vascular length density measured by OCT angiography. (**c**) Representative images of OCT and GCL volume measurement, and OCTA in which foveal vascular length density was measured in each group. Black letters show average GCL thickness and red letters show GCL volume. DM, diabetes mellitus; DR, diabetic retinopathy. *P* values of Kruskal–Wallis test comparing 3 groups were (**a**) 0.008, (**b**) < 0.001. **P < 0.01 by Mann–Whitney U test, comparing 2 groups.

Next, we analyzed the morphological changes using OCT and OCTA. The macular volume of the ganglion cell layer (GCL) in diabetic eyes, both with DR (*P* = 0.007) and without DR (*P* = 0.006), was lower than that in control eyes (Fig. 2a). Moreover, the foveal vascular length density of the superficial retinal layer of DR eyes was lower than that of both control eyes and diabetic eyes without DR (*P* < 0.001 for both) (Fig. 2b). Representative images of OCT and GCL volume measurement, and OCTA in which foveal vascular length density was measured in each group were shown (Fig. 2c).

**Figure 3:**
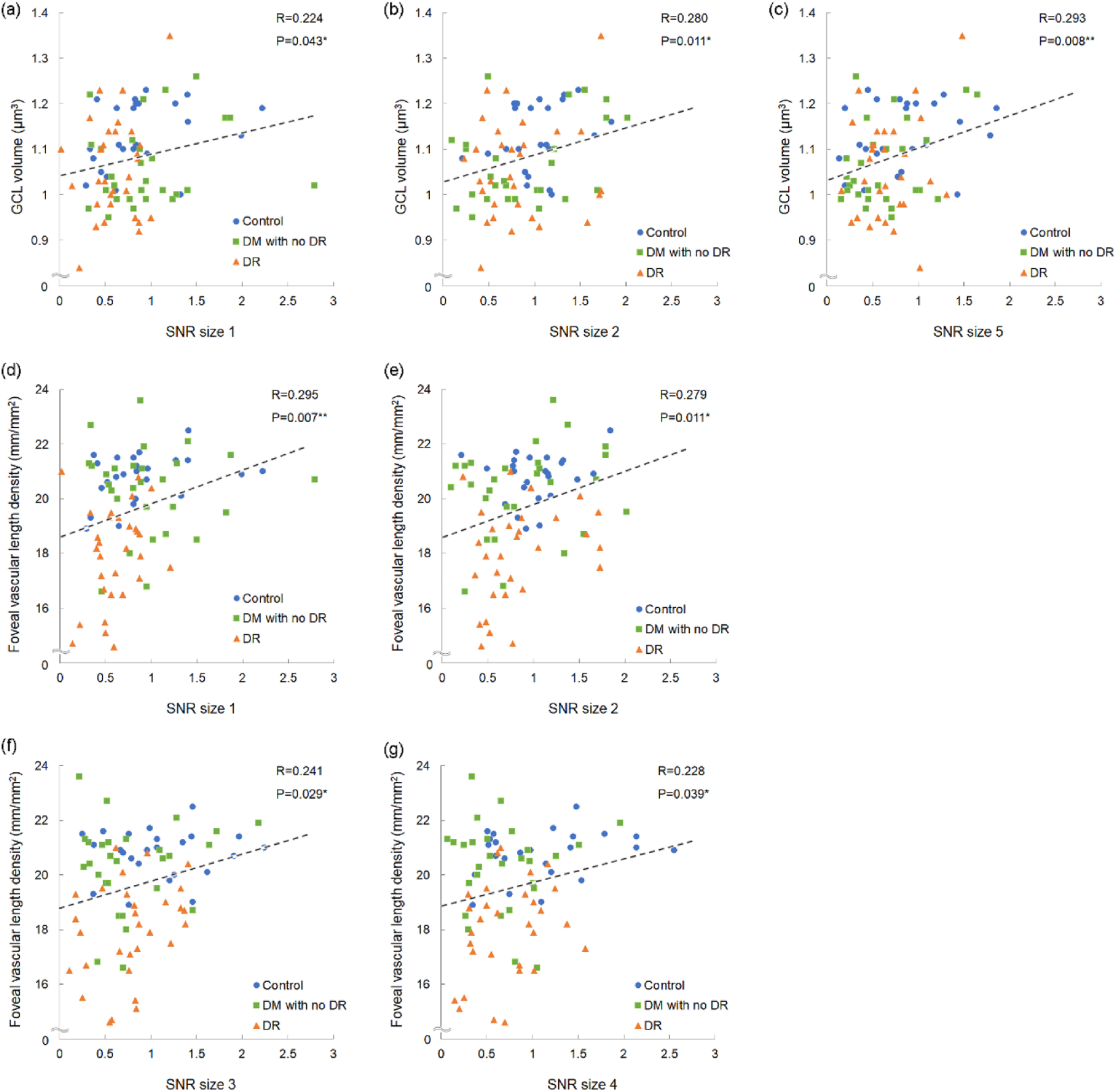
Scatter plots. Scatter plots of the signal to noise (SNR) ratios and macular volume of the ganglion cell layer measured by optical coherence tomography (**a–c**), and foveal vascular length density measured by optical coherence tomography angiography (**d–g**). Pearson’s Correlation Coefficient. **P < 0.05, *P < 0.01.

Finally, the correlations between morphological parameters and visual function as assessed based on swpPERG SNRs was analyzed. GCL volume was found to be correlated with SNRs for stimuli sizes 1 and 2 (*P* < 0.05 for both) as well as 5 (*P* < 0.01) (Table 2). Correlations were also observed between foveal vascular length density and SNRs for stimuli sizes 1 (*P* < 0.01), 2, 3, and 4 (*P* < 0.05 for all) (Table 3). Scatter plots of the data for stimuli sizes with significant results were shown (Fig. 3).

**Table 2:** Correlations between signal to noise ratios and macular volume of the ganglion cell layer measured by optical coherence tomography.

SNR	R	*P*	95% CI
Size 1	0.224	0.043*	0.007 to 0.421
Size 2	0.280	0.011*	0.067 to 0.469
Size 3	0.194	0.081	-0.024 to 0.394
Size 4	0.183	0.101	-0.036 to 0.384
Size 5	0.293	0.008**	0.081 to 0.480
Size 6	-0.046	0.682	-0.260 to 0.173

Pearson’s correlation coefficient.

**P < 0.05; *P < 0.01.

**Table 3:** Correlations between signal to noise ratios and foveal vascular length density measured by optical coherence tomography angiography.

SNR	R	P	95% CI
Size 1	0.295	0.007**	0.083 to 0.481
Size 2	0.279	0.011*	0.066 to 0.468
Size 3	0.241	0.029*	0.025 to 0.435
Size 4	0.228	0.039*	0.012 to 0.424
Size 5	0.163	0.144	-0.056 to 0.367
Size 6	-0.056	0.615	-0.270 to 0.163

Pearson’s correlation coefficient.

**P < 0.05, *P < 0.01.

## Discussion

We demonstrated that swpPERG SNRs could reflect the differences in visual function among control eyes and eyes of patients with diabetes with and without DR. GCL volume in diabetic eyes, both with and without DR, was lower than that in control eyes, and foveal vascular length density was lower in DR eyes than in control eyes and diabetic eyes without DR. Moreover, these morphological parameters were correlated with the visual functional parameter, that is, swpPERG SNR.

The SNRs of not only diabetic DR eyes, but also diabetic eyes without DR, were smaller than those of controls, indicating that visual function was impaired only in the presence of diabetes, irrespective of the presence or absence of visible DR findings on binocular fundus examination. Moreover, the reductions in SNRs and visual function was more severe in eyes with DR than in diabetic eyes without DR. All patients with diabetes had a logMAR BCVA better than 0.05 (equivalent to 0.9 in the decimal score, which corresponds to a value between 20/25 and 20/20 on the Snellen chart). Thus, although all patients had good visual acuity as measured using conventional charts, significant differences in visual function were detected using swpPERG.

We have previously reported that swpPERG can be used to detect subtle changes and variations in visual function among healthy adults with no diagnosed eye diseases, and that stimuli of medium to thin sizes can be used to detect differences among healthy adults more sensitively; SNRs corresponding to thinner size stimuli varied even under healthy conditions, while those for thicker stimuli did not, most likely because everyone can respond well to the latter^14^. Interestingly, differences between diabetic eyes with and without DR were not detected using medium size stimuli, but were detected using thicker size stimuli, suggesting that diabetic eyes without DR were already in an unhealthy condition, and they did not have good SNRs in response to medium size stimuli, as compared to DR eyes, in response to sizes 3 and 4 stimuli. In contrast, the SNR corresponding to size 1 stimuli was significantly smaller in DR eyes than in diabetic eyes without DR, suggesting that DR eyes had inferior visual function and could not achieve comparable scores even with the thickest size stimulus, which would be easier to respond to, compared to thinner size stimuli, although the logMAR BCVA was not different from or inferior to that of diabetic eyes without DR.

GCL volume reduction compared to that in controls was significant in both diabetic eyes with and without DR, suggesting that ganglion cell number was reduced due to diabetes irrespective of DR which is diagnosed based on vascular lesions. This is consistent with the observation that ganglion cell loss was found to be induced by oxidative stress in a mouse model of diabetes^6^. The influence of diabetes on RGCs has also been reported clinically; the amplitude of the photopic negative responses (PhNRs) measured using conventional ERG was found to be reduced in early-stage DR^18^. Meanwhile, the difference in RGC volume between diabetic eyes with and without DR in the current study was not clear. Whether this could be related to the accumulation of pathological material and/or fluid or slight macular edema in eyes with DR is a topic for future research.

Foveal vascular length density was also significantly reduced in eyes with DR compared to that in control eyes and diabetic eyes without DR. The finding of vascular loss in DR is consistent with a previous report^19^. However, the density in control eyes and diabetic eyes without DR was comparable, and no clear vascular loss in diabetic eyes without DR was observed in the current study. Taken together with the GCL volume results, this suggests that neuronal loss may have occurred before obvious vascular loss. DR has long been considered a microvascular disease^20^, and a previous report showed that vascular density was correlated with the oscillatory potentials of scotopic ERG, but not with the PhNR and RGC function^21^. However, more recent studies have suggested that neurodegeneration is an early event^22–25^. Moreover, in one animal model study, retinal vascular networks failed to develop in the absence of RGCs in a non-diabetic background^26^. The current results may support the hypothesis that neuronal loss precedes vascular loss in DR. Conversely, the differences in the results may be related to the detection thresholds of OCT and OCTA. It would be difficult to determine which is the first event during DR development, however, there would be no doubt that neural-vascular interactions progress the retinopathy^27,28^.

Overall, the SNRs were found to correlate well with RGC volume and superficial retinal vascular density. In other words, swpPERG SNRs may reflect the overall condition of the retinal neural tissue involving both neural and vascular systems, which would be an advantage of this system. Nevertheless, this study has some limitations. The sample size was small, gender balance was not similar in each group, while whether there is a gender difference in the swpPERG data would be studied in the future and currently not identified. Skin electrodes, which are placed at longer distance from the retina compared with corneal electrodes, were used, although recent reports have shown the validity in clinical usage in contrast to some of the previous reports^29,30^. Moreover, using skin electrodes was less invasive. Diagnosis of DR was based on a clinical fundus examination and not by fundus photographs and DR levels were not subdivided. Furthermore, some of the diabetic eyes had a BCVA of 0.9 in the decimal score, while that of all control eyes was 1.2 (which corresponds to approximately 20/15 on the Snellen chart).

In conclusion, swpPERG SNRs could reflect fine changes in visual function in diabetic eyes with and without DR, for which the BCVA, as assessed based on conventional charts, had not declined. These subtle visual function changes are most likely related to fine morphological changes in the diabetic retina. The findings of this study provide new insights into the possible applications of swpPERG for evaluating fine differences in visual function in patients with eye diseases, although further studies are required to validate these results. This study may be useful for determining fine changes in visual function in patients with diabetes, for demonstrating the neuroprotective effects of new therapeutic approaches in future clinical trials, and for understanding the pathogenesis of DR.

## Methods

### Participants

The study adhered to the tenets of the Declaration of Helsinki and was approved by the St. Luke’s International University Ethics Committee (approval number 20-RK058). All participants provided written informed consent for the use of their data for research purposes.

Twenty-eight eyes (19 from men) of 20 patients with diabetes but no DR, 30 eyes (25 from men) of 18 patients with diabetes and DR including 12 eyes of 8 patients with proliferative DR, and 24 eyes (4 from men) of 12 healthy, age-matched participants were analyzed in this study. Only eyes with a BCVA better than 0.9 (logMAR 0.05) and no lesions involving the macula were included. Eyes with an axial length more than 27.0 mm and/or diabetic macular edema or macular epiretinal membrane, with or without a history of treatment, were excluded.

### Eye examinations

BCVA was measured based on refraction tests, and the decimal scores measured using the Landolt C chart were converted to the corresponding logMAR values. Slit-lamp examination and binocular indirect ophthalmoscopy were performed after pupil dilation using 0.5% tropicamide. The axial length was measured using an IOL-Master 500 (Carl Zeiss Meditec AG, Jena, Germany) optical biometer. The diagnosis of DR was done by binocular indirect ophthalmoscopy by a retinal specialist (YO).

### swpPERG

swpPERG recordings were obtained using the EvokeDx (Konan Medical, California, USA) visual electrophysiology system as previously reported^14^. Briefly, spatial-patterned (horizontal grating) and contrast-reversed stimuli of sizes 1 to 6 were displayed (Supplementary Figure S1), and the corresponding data were obtained through dual-channel-amplifier under photopic conditions using skin electrodes according to the manufacturer’s protocol. Briefly, 5 skin electrodes (carbon electrodes) were placed at each inferior eye lid (active), 2 cm from each outer canthus (reference) and forehead (ground) (Supplementary Figure S2). The 17-inch display monitor was placed at a distance of 65 cm from the participants. If the participants needed correction for BCVA measurement, they used contact lenses during the examination. The ERG responses for 2 s were recorded, and decomposed to constituent frequency components at 10, 20, 30, 40, 50, and 60 Hz (Supplementary Figure S3). Fourier filtering technique was used to remove excessive environmental noise at 60 Hz. The frequency component after stimulation by each stimulus size was compared with the background brain activity recorded by electroencephalography as noise, and the data were expressed as the SNRs. The value at 10 Hz of the Fourier transformed data was used in the current study according to the manufacture’s recommendation. The processes were performed in the built-in software.

### OCT and OCTA

Sectional and three-dimensional OCT images obtained using spectral-domain OCT (Spectralis OCT, Heidelberg Engineering GmbH, Dossenheim, Germany) were analyzed using the built-in device software to measure central retinal thickness and macular volumes according to the Early Treatment Diabetic Retinopathy Study (ETDRS) grid of retinal layers; 6 mm diameter areas were analyzed. OCTA images recorded using spectral-domain OCT (CIRRUS 5000, Carl Zeiss Meditec, AG) were analyzed using the built-in device software to measure the foveal vascular length density at the superficial layer in 3 mm diameter areas.

### Statistical analysis

Data are presented in terms of median (range) values. The Kruskal–Wallis test, Mann–Whitney test, and Pearson’s correlation coefficient method as well as Shapiro–Wilk test were used to analyze the data. IBM SPSS Statistics, version 24.0 (Armonk, NY, IBM Corp.) was used for all statistical analyses. *P* values < 0.05 were considered statistically significant.

### Ethical approval

This retrospective study adhered to the tenets of the Declaration of Helsinki, was approved by the St. Luke’s International University Ethics Committee (approval number: 20-RK058), and registered as UMIN000040444.

### Consent to participate and consent to publish

This retrospective study was approved by the St. Luke’s International University Ethics Committee (approval number: 20-RK058).

### Supplementary Information

Supplementary Information.

## Data Availability

The datasets generated during and/or analyzed during the current study are available from the corresponding author on reasonable request.
