# Supplementary material for: Evaluating fine changes in visual function of diabetic eyes using spatial-sweep steady-state pattern electroretinography
Source: Sci Rep. 2023 Aug 22;13:13686. doi: 10.1038/s41598-023-40686-5 (PMC10444753; doi:10.1038/s41598-023-40686-5)
Supplement: Supplementary file 1 — Supplementary Information. [file 41598_2023_40686_MOESM1_ESM.pdf]

Evaluating fine changes in visual function of diabetic eyes using spatial-sweep steady-state pattern electroretinography

Norihiro Nagai[1,2], Yasuaki Mushiga1,2, and Yoko Ozawa[1,2,3]*

[1]Department of Ophthalmology, St. Luke's International Hospital
[2]Department of Ophthalmology, Keio University School of Medicine
[3]Department of Clinical Regenerative Medicine, Eye Center, Fujita Medical Innovation Center Tokyo, Fujita Health University School of Medicine

Running head: Fine changes of visual function in diabetic eyes

*Correspondence author
Yoko Ozawa M.D., Ph.D
Professor
Department of Clinical Regenerative Medicine
Eye Center, Fujita Medical Innovation Center Tokyo
Fujita Health University School of Medicine
7-16-14 Ginza, Chuoku, Tokyo 104-8313
Tel; +81-3-6450-1092
ozawa@a5.keio.jp, yoko.ozawa@fujita-hu.ac.jp
ORCID: 0000-0003-4797-5705

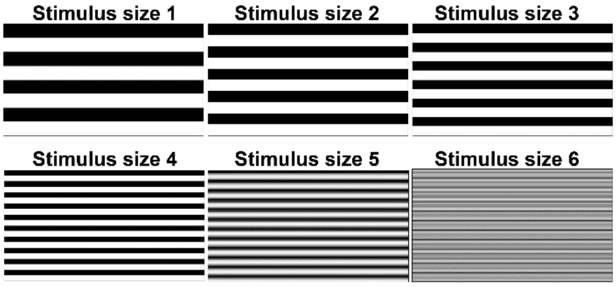

Supplementary Figure S1. Stimulus sizes 1 to 6.

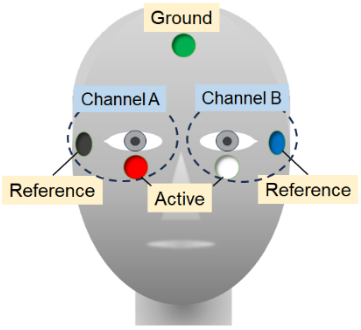

Supplementary Figure S2. Electrode positioning.
Five skin electrodes were placed at each inferior eye lid (active), 2 cm from each outer canthus (reference) and forehead (ground).

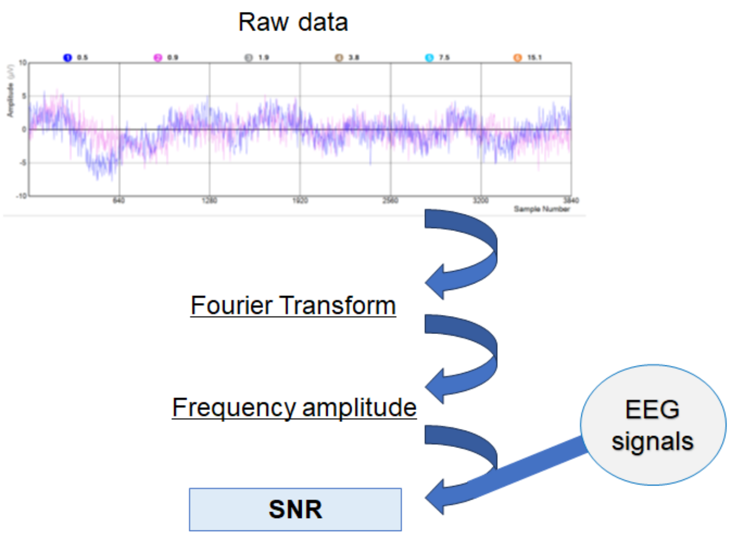

Supplementary Figure S3. System of the spatial-sweep steady-state pattern electroretinography.
